# Increased inflammatory cytokines and oxidative stress enhanced antibody production in breast and prostate cancer patients with COVID-19 related depression

**DOI:** 10.3389/fchem.2023.1192074

**Published:** 2023-04-20

**Authors:** Naif K. Binsaleh, Subuhi Sherwani, Reem Eltayeb, Husam Qanash, Abdulrahman S. Bazaid, Maryam Althobiti, Mohannad S. Hazzazi, Saravanan Rajendrasozhan

**Affiliations:** ^1^ Department of Medical Laboratory Sciences, Faculty of Applied Medical Sciences, University of Ha’il, Ha’il, Saudi Arabia; ^2^ Department of Biology, Faculty of Sciences, University of Ha’il, Ha’il, Saudi Arabia; ^3^ Department of Clinical Laboratory Science, College of Applied Medical Science, Shaqra University, Shaqra, Saudi Arabia; ^4^ Department of Medical Laboratory Sciences, Faculty of Applied Medical Sciences, King Abdulaziz University, Jeddah, Saudi Arabia; ^5^ Hematology Research Unit, King Fahd Medical Research Center, King Abdulaziz University, Jeddah, Saudi Arabia; ^6^ Department of Chemistry, Faculty of Sciences, University of Ha’il, Ha’il, Saudi Arabia

**Keywords:** breast cancer, prostate cancer, COVID-19, depression, oxidative stress, antibody, inflammatory cytokines

## Abstract

Cancer management is highly dependent on the immune status of the patient. During the COVID-19 pandemic, a large number of people suffered from anxiety and depression, especially cancer patients. The effect of depression on breast cancer (BC) and prostate cancer (PC) patients, during the pandemic has been analyzed in this study. Levels of proinflammatory cytokines (IFN-γ, TNF-α, and IL-6) and oxidative stress markers malondialdehyde (MDA) and carbonyl content (CC) were estimated in patients’ serum samples. Serum antibodies against *in vitro* hydroxyl radical (^•^OH) modified pDNA (^•^OH-pDNA-Abs) were estimated using direct binding and inhibition ELISA. Cancer patients showed increased levels of proinflammatory cytokines (IFN-γ, TNF-α, and IL-6) and oxidative stress markers (MDA and CC levels), which were further significantly enhanced in cancer patients with depression compared to normal healthy (NH) individuals. Increased levels of ^•^OH-pDNA-Abs were detected in breast cancer (0.506 ± 0.063) and prostate cancer (0.441 ± 0.066) patients compared to NH subjects. Serum antibodies were found to be significantly elevated in BC patients with depression (BCD) (0.698 ± 0.078) and prostate cancer patients with depression (PCD) (0.636 ± 0.058). Inhibition ELISA also exhibited significantly high percent inhibition in BCD (68.8% ± 7.8%) and PCD (62.9% ± 8.3%) subjects compared to BC (48.9% ± 8.1%), and PC (43.4% ± 7.5%) subjects. Cancer is characterized by enhanced oxidative stress and increased inflammation, which may be exaggerated with COVID-19 related depression. High oxidative stress and compromised antioxidant homeostasis exerts alterations in DNA, leading to formation of neo-antigens, subsequently leading to the generation of antibodies. COVID-19 pandemic related depression needs to be addressed globally for improved cancer patient care and cancer disease management.

## 1 Introduction

Cancer is a chronic inflammatory disease and one of the leading causes of death worldwide. According to the latest data by WHO (world Health Organization), one in eleven females and one in eight males died due to cancer worldwide in 2022 (World Health Organization, 2020). During the COVID-19 pandemic, due to burden on the healthcare system, many cancer patients suffered delays in treatment, arising from limited access to necessary medical facilities.

At the start of 2020, the severe acute respiratory syndrome coronavirus 2 (SARS-CoV-2) spread around the world at a very fast pace, and was declared a pandemic by the WHO (Cucinotta and Vanelli, 2020). The pandemic caused a negative impact on social life styles, healthcare systems and economic conditions of individuals worldwide leading to distress, stress and anxiety (Choi et al., 2020; Daly and Robinson, 2022). Infected individuals present mild to severe symptoms, which may include respiratory distress, and in severe cases multi-organ failure or even death. The primary reason for inflammatory changes is the excessive production of cytokines (Sherwani and Khan, 2020). Extended periods of lockdown, social distancing norms and other mandatory protective practices at home and in the workplace impacted levels of physical activity as well as the mental health of individuals, subsequently leading to anxiety and depression among a large population (Trabelsi et al., 2021; van der Werf et al., 2021). Several studies have already been published on depression during unprecedented COVID-19 pandemic (Wan Mohd Yunus et al., 2021; Bueno-Notivol et al., 2021). Depression rates were found to increase in several countries impacting the mental health status of populations (Bueno-Notivol et al., 2021). All age groups were affected due to an overall increase in physiological stress, fear, anxiety and depression (Rodríguez-Hidalgo et al., 2020; Saraswathi et al., 2020).

The burden of a cancer diagnosis itself causes immense psychological and physiological stress in patients, which might have been further aggravated due to the stress and uncertainty of the pandemic too. Increased oxidative stress is found in cancer patients as well as in depressed individuals. Increased generation of reactive species cause non-repairable damage in cells, causing cell death (Hussain et al., 2016). Inflammatory cytokines directly and indirectly play an important role in enhancing oxidative stress and through multiple pathways support tumor cell progression (Kartikasari et al., 2021). Extensive production of reactive species and disease induced redox homeostasis imbalance (Hussain et al., 2016) may cause alterations and/or degradation of biomolecules (DNA, proteins, lipid etc.)

Taking all these into account, this study has been designed to analyze the inflammatory and oxidative stress, as well as antibodies against hydroxyl radical (^•^OH) treated plasmid-DNA in males with BC and PC patients alone or with depression (BCD and PCD). The estimation of humoral immune status in these cancer patients with or without COVID-related depression might provide an insight into the immune imbalance associated with COVID-19 and better cancer management and palliative care.

## 2 Materials and methods

Complete and incomplete Freund’s adjuvant, Dialysis tubing cellulose membrane pUC18 plasmid DNA, hydrogen peroxide, PBS solution, catalase, mannitol, EDTA, SOD, IgG-alkaline phosphatase conjugate, p-nitrophenyl phosphate (Aldrich Sigma, United States). Cytokine kits (IL-6, IFN-γ and TNF-α) were purchased from R&D Systems, Minneapolis, MN, United States.

### 2.1 Free radical modification of plasmid DNA

Plasmid DNA (1 mg/mL) was treated with hydrogen peroxide (10 mM) and the reaction mixture was exposed to UV light at 254 nm for 30–45 min at room temperature under dark conditions. The reaction was carried out in 10 mM PBS solution at pH 7.4. The reaction induced formation of hydroxyl radicals and the reaction mixture was dialyzed against PBS solution to remove unwanted free radicals. The resultant modified plasmid DNA was stored at −80°C for further experiments (Khan et al., 2011).

### 2.2 Analysis of pDNA modification by spectroscopic studies

UV analysis: Both native and ^•^OH modified pDNA were screened under UV-spectrophotometer (UV-1700, Shimadzu, Kyoto Japan) to determine change in UV intensities at 260 nm wavelength. Quenching studies using catalase (500 units/mL), mannitol (100 mM), EDTA (100 mM) and SOD (500 units/mL) were performed for the changes in the percent modifications of UV intensities at 260 nm.

#### 2.2.1 Thermal stability

Native and modified pDNA were analysed for their stability. All the samples were treated with varying temperatures ranging from 30°C to 96°C. UV spectrophotometer was connected with a thermal chamber to increase the temperature of the sample at a rate of 1.0°C/min. All absorbance was analysed and recorded at 260 nm.

#### 2.2.2 Fluorimetry

Fluorescence intensities were observed for both native and modified pDNA. A wavelength range of 350–600 nm was used to record the fluorescence spectra for both samples on spectrofluorometer (RF-5301, Shimadzu, Tokyo Japan) (Binsaleh et al., 2022).

### 2.3 Sera samples collection

Nintey sera samples were collected from patients and normal healthy subjects from June 2021—December 2021. The research study was conducted according to the Declaration of Helsinki (1964). Serum samples collected from normal healthy male (*n* = 15) and female (*n* = 15) individuals were from individuals who did not show any signs or symptoms of any disease. Fifteen serum samples were collected for each of the following groups; prostate cancer (PC), breast cancer (BC), prostate cancer with depression (PCD), and breast cancer with depression (BCD). Samples were collected from volunteers with their full written consents as per the approval of the Research Ethics Committee at the University of Ha’il; study protocol H-2021-122. Patients and healthy controls were checked using similar diagnostic procedures and the disease was confirmed by an oncologist based on the recommended procedures for prostate cancer (e.g., physical examination, antigen tests, and histopathology analysis, etc.) and breast cancer (e.g., physical examination, antigen tests, mammography, histopathology, etc.). All cancer patients included in this study exhibited metastasis at the time of diagnosis.

Demographic (age and gender) and clinical data such as fasting blood glucose (FBG), glycated hemoglobin (HbA1c), basal metabolic index (BMI), C-reactive protein (CRP)) data fasting blood glucose (FBG), glycated hemoglobin (HbA1c), basal metabolic index (BMI), C-reactive protein (CRP) etc. were also collected. FBG, HbA1c, and BMI were analyzed by the recommended prescribed procedures applied in the diagnostic clinics. Levels of CRP were measured using the latex agglutination reaction method.

Volunteers were completed a modified version of a self rating questionnaire to screen the individuals and determine their level of depression (Kroenke and Spitzer, 2002). The depression index score was evaluated as the total score from 20 questions divided by 80 (the maximum possible score). The questionnaire was discussed with a physiatrist in the College of Medicine.

Serum samples were isolated from blood collected (3–5 mL) from volunteers and serum complement proteins were deactivated by heating the samples at 56°C for half an hour and then stored at −80°C.

Exclusion criteria for the individuals and patients included individuals aged less then 18 years, pregnancy or lactation, patients using antibiotics, alcohol drinkers and smokers. Individuals with any previous history of COVID-19 disease or associated complications were excluded from this study.

### 2.4 Detection of carbonyl compounds

Amount of protein bound carbonyl was detected in the sera samples of cancer patients and healthy individuals, as described by a published procedure (Levine et al., 1990). Briefly, 100 μL of serum sample was mixed with 400 μL of dinitrophenyl hydrazine (DNPH). Control samples were devoid of DNPH. The mixture was incubated for 1 h at 25°C and then precipitation of DNP-hydrazones was done by adding 500 μL of trichloroacetic acid (4% w/v). The sample was centrifuged for 3–5 min at 13,000 g. To remove the non-reacted DNPH, pellet was dissolved in ethanol-ethylacetate (1:1, v/v) and then centrifuged. The centrifugation was repeated about 3–4 times, and pellet was dissolved in 0.6 mL guanidinium HCl (6M, pH 2.3). To dissolve the hydrazones completely, the sample mixture was frozen at −20 C and thawed. A 200 μL from the aliquot was read at 379 nm using ELISA reader (MR9600-415 Accuris, NJ, United States). The results were evaluated as nanomoles of carbonyl per mg of protein using a ε379 nm = 22,000 M^−1^cm^−1^.

### 2.5 Serum malondialdehyde contents

Serum MDA levels were identified using a comercially available ELISA kit (Elabscience, United States). Lipid oxidative stress was assessed by measuring MDA levels in serum samples. Sampes were analysed as instructed in the ELISA kit manual. Absorbance of test samples was measured at 532 nm on a microplate reader. MDA contents were calculated with the following formula:

$$MDA=\frac{X1}{X2}\times C$$

X_1_; OD of test sample—OD of control.X_2_; OD of standard—OD of blank.C; Standard concentration (10 nmol/mL).

### 2.6 Cytokine estimation

Detection and analysis of levels of inflammatory cytokines IL-6, IFN-γ and TNF-α in serum samples of cancer patients and NH subjects, was done using quantitative ELISA (sandwich) (R&D System, Minneapolis, MN, United States), with a sensitivity of less than 0.5 pg/mL for all cytokines. Each sample was assayed in three different wells.

### 2.7 Antibodies raised against native and ^•^OH-pDNA

Antibodies against native and ^•^OH-pDNA were raised in female rabbits as mentioned previously (Alouffi et al., 2018; Khan et al., 2019a). Briefly, first dose of antigens (50 μg) were emulsified with equal volume of Freund’s adjuvant (complete), and injected intramuscularly into the experimental animals. Subsequently, similar amount of antigens (50 μg) mixed with equal volume of Freund’s adjuvant (incomplete) were injected into the animals. A dose of about 400 μg antigens was received by each animal. Blood samples were collected regularly, and serum samples were separated as mentioned above and stored at −80°C. Blood samples were also collected before immunization as pre-immune sera to be used as negative controls.

### 2.8 Serum IgG purification

Pre-immune and immunized experimental animals serum samples were used to isolate purified IgGs using Protein-A Agarose column (Sigma-Aldrich, United States) by using a published procedure (Goding, 1978). A ratio of 1:1 (1 mL) serum sample and phosphate buffer saline (pH 7.4) were applied to Protein-A Agarose column (5 mL). Then column was cleaned 2–3 times with PBS buffer to remove unbound IgG. Acetic acid (0.6%) and NaCl (0.9%) were used to elute the bound IgGs from the column. Eluted samples were neutralized by adding 1 mL of Tris-HCl (1M, pH 8.5). Eluted fractions were analyzed on a spectrophotometer at wavelength of 251 nm and 278 nm, to calculate the concentration of IgG. The optical density of 1.4 at 280 nm is equivalent to 1.0 mg/mL of purified IgG.

### 2.9 Enzyme-linked immunosorbent assay

Direct binding ELISA was performed to detect the presence of antibodies against native and pDNA in healthy individuals and patients’ sera (Sherwani et al., 2022; Sherwani and Khan, 2022). Levels of antibodies against both native and modified pDNA were screened in immunized animals, as described elsewhere (Khan et al., 2020).

Competition ELISA was also done to evaluate recognition of specific antibodies from healthy control and cancer patient sera samples, as well as immunized antibodies to native and modified pDNA (Khan et al., 2020; Binsaleh et al., 2022; Sherwani et al., 2022). Briefly, both the antigens (pDNA and ^•^OH-pDNA) at a concentration of 5 μg/mL were added to the 96 well ELISA plates and kept at room temperature for 3–4 h. ELISA plates were washed with TBS-T (Tris Buffer Saline with Tween 20) and plates were blocked with bovine serum albumin (1%). Immune complexes, formed in the test tubes using sera samples (1:100 dilutions) and varying concentrations (0–10 μg/mL) of antigens, were added and incubated for 4 h and then overnight in a refrigerator at 4°C. These immune complexes (100 µL) were transferred to the ELISA plates and incubated at room temperature for 3–4 h. ELISA plates were washed with TBS-T and anti-human IgG-alkaline phosphate conjugate was added for 1 h and again ELISA plates were washed. Then the substrate p-nitrophenyl phosphate was added, and within 20 min the microplates were read at 410 nm, using a microplate reader (MR9600-415 Accuris, NJ, United States).

### 2.10 Statistical analysis

Results are presented as mean ± SD. Normality tests and multiple comparison were performed using a software SPSS (16.0, Chicago, United States). Significance was calculated using the Student’s t-test. All significances were represented as *p*-value of <0.05.

## 3 Results

### 3.1 Clinical characterization

Clinical investigations were carried out for patients with cancer alone or with depression as well (Table 1). There was slight increase in FBG and HbA1c levels in cancer patients compared to their respective healthy controls, however, the changes were not significant. Similarly, BMI and waist-to-hip ratios of cancer patients were found to be slightly higher than the healthy controls. No significant differences were observed in FBG, HbA1c, BMI and waist-to-hip ratios in patients with cancer alone or with depression.

**TABLE 1:** Clinical and demographic presentation of cancer and healthy subjects.

Groups	Gender	Age	Menopausal status (number)	Disease duration	Fasting blood glucose (mg/dL)	HbA1_C_ (%)	BMI (kg/m^2^)	Waist-to-hip ratio
	M/F							
NH-M	15	48 ± 12.1	—	—	84.3 ± 7.1	5.34 ± 0.08	23.61 ± 2.87	0.78 ± 0.073
NH-F	15	47 ± 11.7	8	—	85.5 ± 6.3	5.37 ± 0.09	23.21 ± 2.93	0.79 ± 0.076
Prostate cancer	15	49 ± 12.6	—	8 ± 4.1	87.1 ± 8.5	5.53 ± 0.11	24.11 ± 3.04	0.81 ± 0.074
Breast cancer	15	48 ± 13.3	7	7 ± 3.6	86.9 ± 8.5	5.51 ± 0.13	24.91 ± 2.93	0.82 ± 0.079
Prostate cancer with depression	15	58 ± 14.5	—	9 ± 4.5	87.8 ± 8.4	5.55 ± 0.12	25.11 ± 3.09	0.81 ± 0.073
Breast cancer with depression	15	51 ± 16.3	6	8 ± 4.7	88.8 ± 7.6	5.53 ± 0.13	26.71 ± 2.98	0.82 ± 0.078

All the patients included were of metastasis stage at the time of diagnosis. All the patients were on chemotherapy with other medications. Subcategories of any cancer is not defined. Each sample was run in triplicate. All data is given as mean ± standard deviation (SD).

Furthermore, clinical analyses were conducted for the sera samples of prostate and breast cancer patients for CRP, cytokines levels (IFN-γ, TNF-α and IL-6), and oxidative stress level markers (MDA and carbonyl contents) (Table 2). A significant increase (*p* < 0.001) in the levels of CRP was observed in prostate and breast cancer patients, which was further enhanced with accompanying depression. Also, a substantial increase (*p* < 0.001) in the levels of IL-6 was observed in prostate and breast cancer patients when compared to the healthy controls. However, no respectable difference in the IL-6 levels was observed in patients with cancer alone or cancer with depression. In contrast, there was a significant increase in the levels of IFN-γ and TNF-α in cancer patients with depression compared to the cancer patients (postrate and breast cancer) alone.

**TABLE 2:** Oxidative stress, inflammatory marker, and inflammatory cytokines analysis of cancer patients and healthy subjects.

Groups	MDA (nmol/mL)	Carbonyl content (nmol/mg protein)	C-reactive protein (mg/L	IFN-γ (pg/mL)	TNF-α (pg/mL)	IL-6 (pg/mL)
HC-M (*n* = 15)	2.61 ± 0.16	0.78 ± 0.084	0.92 ± 0.077	3.0 ± 0.21	0.94 ± 0.09	1.97 ± 0.16
HC-F (*n* = 15)	2.58 ± 0.17	0.93 ± 0.089	0.93 ± 0.071	3.2 ± 0.29	0.90 ± 0.11	1.99 ± 0.17
Prostate cancer (*n* = 15)	4.11 ± 0.12*	2.84 ± 0.16*	5.01 ± 0.47***	4.0 ± 0.32*	1.33 ± 0.18*	7.3 ± 0.61***
Breast cancer (*n* = 15)	4.39 ± 0.21*	2.22 ± 0.18*	5.24 ± 0.54***	4.2 ± 0.29*	1.39 ± 0.13*	7.1 ± 0.65***
Prostate cancer with depression (*n* = 15)	5.41 ± 0.19**	3.64 ± 0.25**	7.13 ± 0.66***	4.9 ± 0.30**	2.73 ± 0.21***	7.5 ± 0.62***
Breast cancer with depression (*n* = 15)	5.97 ± 0.23**	3.92 ± 0.23**	7.67 ± 0.62***	5.3 ± 0.35**	2.99 ± 0.22***	6.8 ± 0.59***

Each sample was run in three different wells. Values are presented as mean ± standard deviation. *p < 0.05, **p < 0.01, ***p < 0.001. Statistical values were evaluated by comparing healthy controls with gender matched patients.

Oxidative stress markers were estimated in the serum samples to understand the patients’ oxidative stress levels. Postrate and breast cancer patients exhibited significantly higher levels of MDA and carbonyl contents compared to the healthy individuals. The levels of these markers were found to further increase in cancer patients with depression (Table 2).

### 3.2 Hydroxyl radical modification of pDNA

Native pDNA was modified using hydroxyl radicals in *in vitro* reaction. Modified pDNA was analyzed for structural alterations due to the presence of ^•^OH radicals. Native and ^•^OH-pDNA were analyzed using spectrophotometer at a wavelength 260 nm. The UV intensity was observed to increase in ^•^OH-pDNA as compared to native pDNA (Table 3). This may be due to the exposure of the nitrogenous bases of the pDNA, upon free radical damage.

**TABLE 3:** Characterization of hydroxyl radical modified plasmid DNA.

Groups	Native p-DNA	^•^OH-pDNA
Absorbance at 260 (37°C)	2.04 ± 0.09	2.88 ± 0.014**
Percent hyperchromacity at 260 nm (95°C)	20.9 ± 0.13	34.7 ± 0.19**
Fluorescence (Exc. 520 nm)	589 ± 8.8 AU	837 ± 11.4***

Significant changes were representing as **p < 0.01; ***p < 0.001. Exc. and nm represent excitation and nanometre. For statistical analysis native p-DNA, values were compared with ^•^OH-pDNA, values.

The stability of the native and modified DNA samples was also subjected to high temperatures (30°C—96°C) and UV intensities of treated samples were observed. Increased fluorescence intensity was recorded at 520 nm after the hydroxyl modification of pDNA (Table 3). This may be due to the breakdown and unwinding of pDNA and exposure of nitrogenous bases.

### 3.3 Quenching studies

**FIGURE 1:**
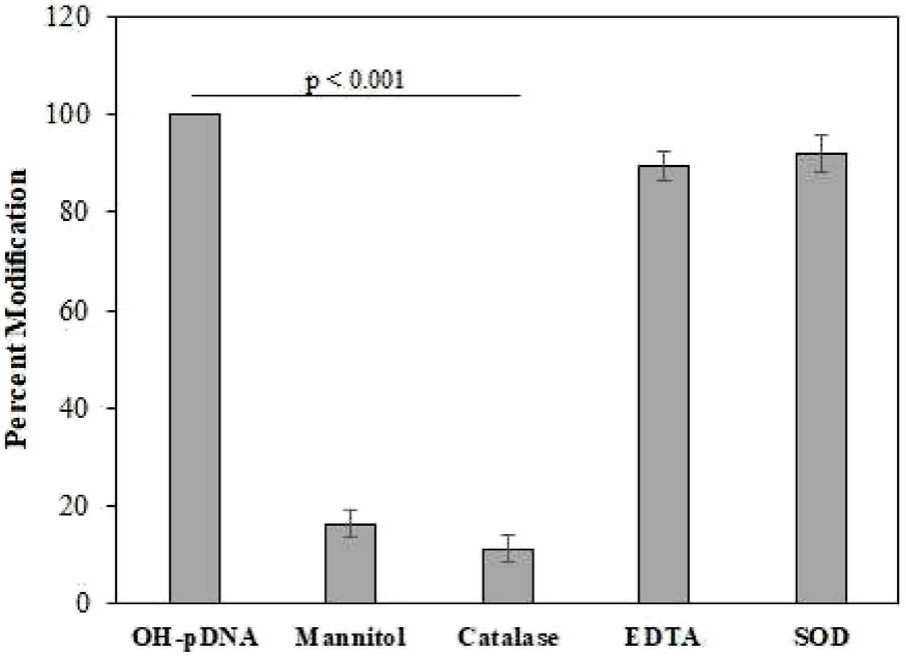
The OH-pDNA was considered as 100% modification based on the UV analysis at 260 nm. Quenching studies were performed using catalase (500 units/mL), mannitol (100 mM), EDTA (100 mM) and SOD (500 units/mL). Antioxidant were co-incubated with the reaction during modification of the pDNA and samples were analysed at 260 nm. All the reactions were carried out in triplicates. Significance presented as *p* < 0.01.

Quenching studies were conducted using various antioxidants (mannitol, catalase, and SOD) and a metal chelating agent (EDTA), used in pDNA modification reaction mixtures (Figure 1). After the reactions all the samples were dialyzed against the PBS and the percent modifications were observed using an Absorbance of 260 nm. The modified sample, devoid of any quenching agent, was considered as positive control (100% modification). Results showed highest decrease in the modification by catalase and mannitol, both specific antioxidants for ^•^OH and H_2_O_2_ molecules. This shows the involvement of H_2_O_2_ and ^•^OH in pDNA modification.

### 3.4 Antigenicity of pDNA and ^•^OH modified pDNA

Antigens pDNA and OH-pDNA were administered to female rabbits for up to 8 weeks (details are provided in the methods section). Blood samples were collected before the administration of antigens (pre-immune sera) and afterwards at different intervals. Immunoglobulin G was isolated from pre-immune and immune rabbit serum samples using Protein A-Agarose column. Purity was assessed for different IgG samples on 7% SDS-polyacrylamide using gel electrophoresis (data not shown).

**FIGURE 2:**
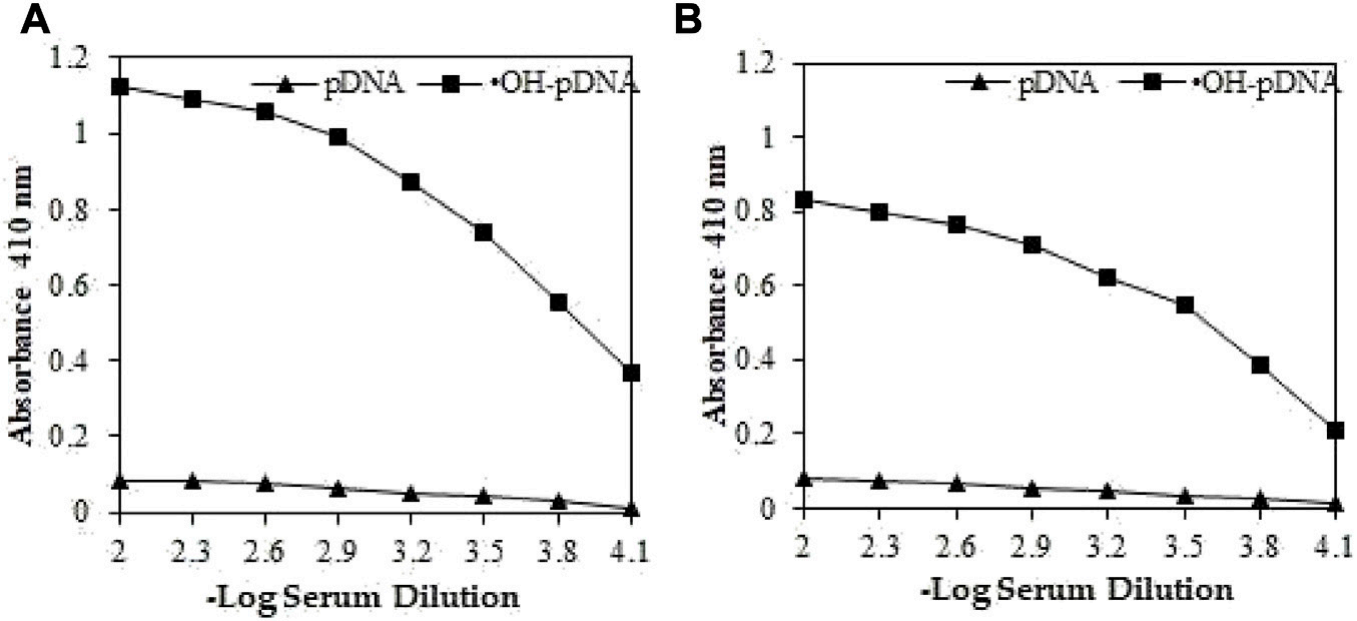
Direct binding ELISA of OH-pDNA **(A)** and native pDNA **(B)** from preimmune (▲) and immune sera (**■**) sera samples from immunized animals. Microtitre plates were coated with 5 μg/mL of respective antigens.

Direct binding ELISA was used to assess the antibody production against respective antigens. IgGs raised against modified p-DNA, exhibited significantly high titre (>1:25,600) (Figure 2A). IgGs raised against native p-DNA also showed high titre (∼1:12,800) (Figure 2B). However, antibody production against the ^•^OH-pDNA showed almost two-fold higher titre than native pDNA in immune sera samples of experimental animals. Pre-immune sera IgG exhibited low number of antibodies against both antigens.

**FIGURE 3:**
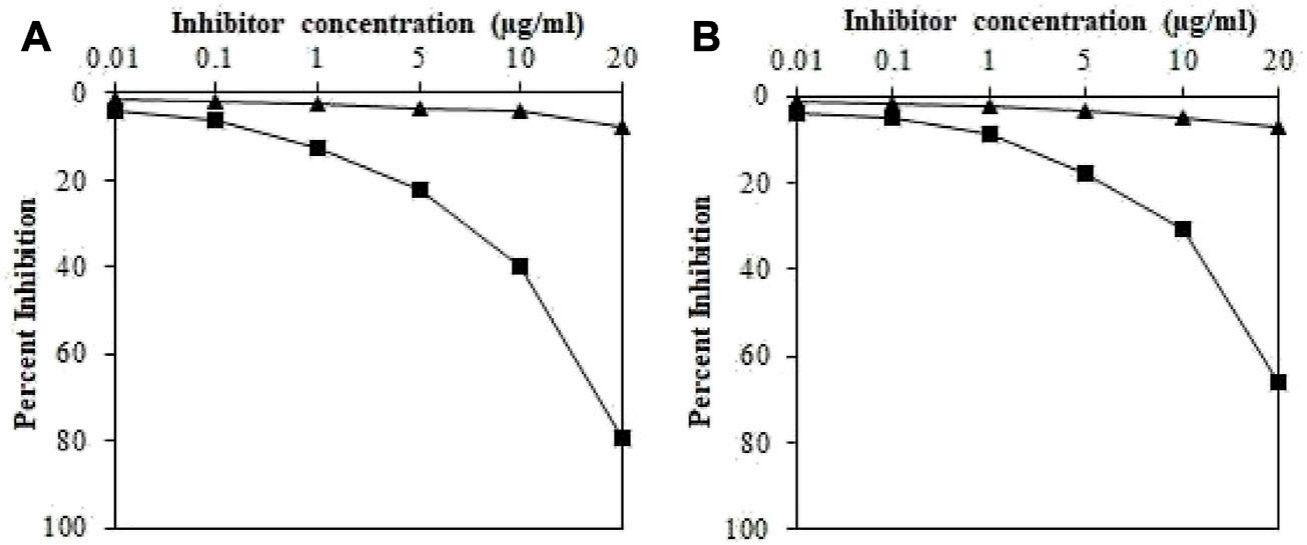
Inhibition ELISA of IgGs raised against OH-pDNA **(A)** and pDNA **(B)** (■) and IgGs from pre-immune sera (▲) samples. The 96-well plates were coated with pDNA and OH-pDNA (5 μg/mL). All the samples were run in triplicates.

Induced antibodies were isolated as mentioned above and specificities were analyzed against their immunogens using inhibition ELISA. The maximum percent inhibition (MPI) for IgGs against ^•^OH-pDNA was determined to be 79.3% at 20 ug/mL (Figure 3A), whereas, IgGs against pDNA showed 66.1% (Figure 3B). Moreover, IgGs against ^•^OH-pDNA showed 50% inhibition at 12.1 ug/mL. However, IgGs against pDNA exhibited 50% inhibition at 17.7 ug/mL of the antigen.

These results indicate high production of antibodies (IgGs) against ^•^OH-pDNA in experimental animals as compared to native pDNA. Raised antibodies were also highly specific for the modified antigen as compared to the native antigen.

### 3.5 Detection of serum antibodies against ^•^OH-pDNA

**FIGURE 4:**
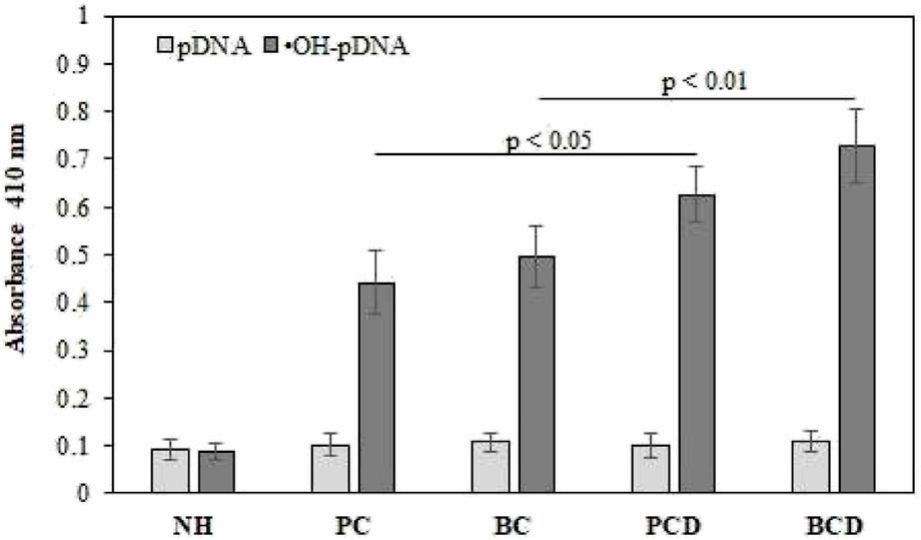
Direct binding ELISA of serum antibodies against native and ^•^OH-pDNA antigen. ELISA plates were coated with the respective antigen (5 μg/mL). All the samples were tested in triplicates. Significance was represent as *p* < 0.05 and *p* < 0.01.

The sera samples (1:100 dilutions) of all subjects were analyzed for the presence of antibodies (IgG) against native and ^•^OH-pDNA antigens (Figure 4). Direct binding ELISA was used to screen for the levels of the antibodies against ^•^OH-pDNA (anti-^•^OH-pDNA antibodies). Direct binding data showed highest binding of serum antibodies against modified antigen in breast cancer patients with depression (OD; 0.698 ± 0.078), followed by prostate cancer patients with depression (0.636 ± 0.058), breast cancer patient alone (0.506 ± 0.063) or prostate cancer patients (0.441 ± 0.066) alone. Normal human subjects did not show respectable binding against either of the antigens.

**FIGURE 5:**
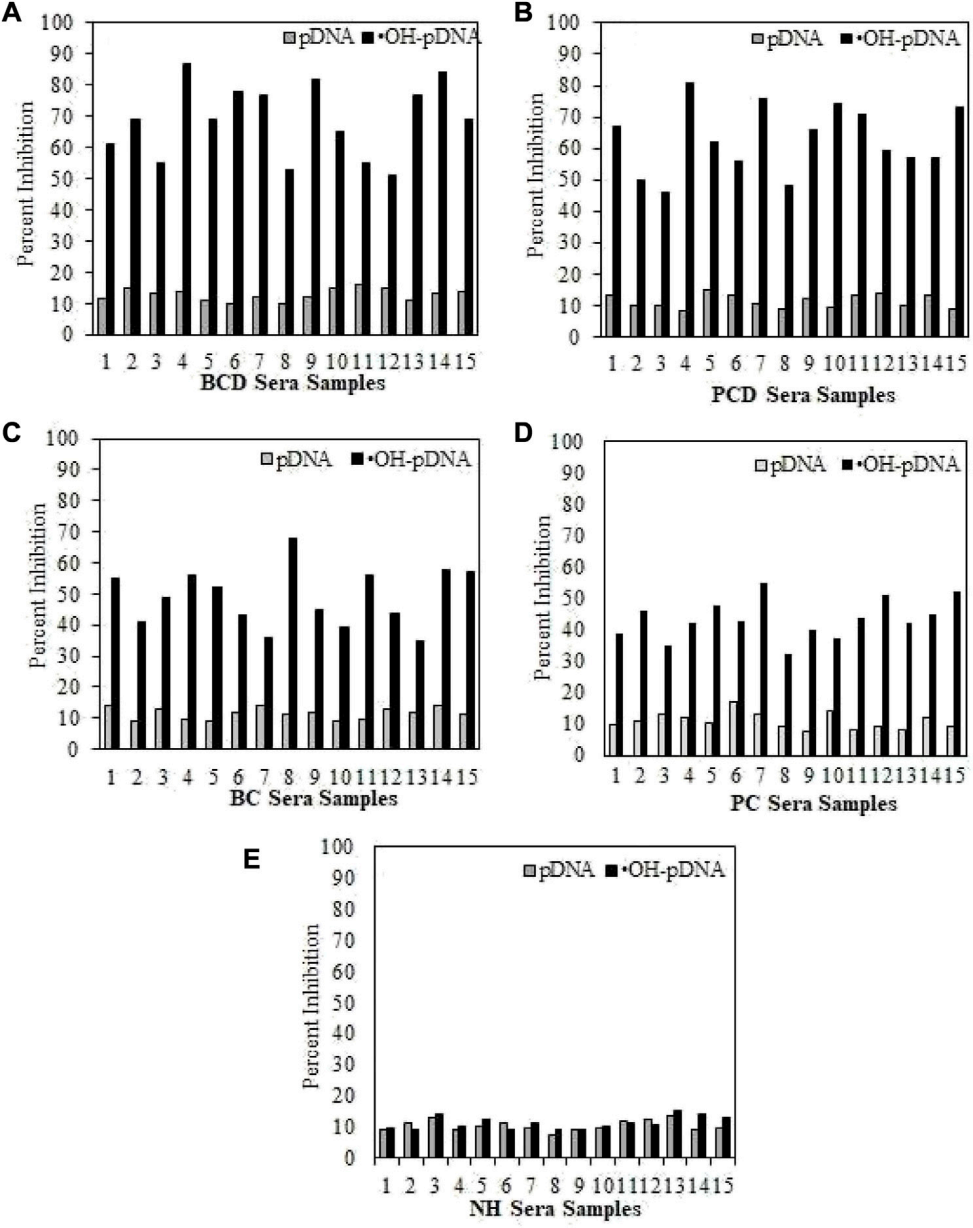
Inhibition ELISA for cancer and normal human subjects against native and modified antigens (10 μg/mL). maximum percent inhibition at 10 μg/mL were assessed for serum samples from BCD **(A)**, PCD **(B)**, BC **(C)**, PC **(D)** and NH serve as controls **(E)**. Each sample was tested in triplicates and the values given are in mean ± SD.

Serum antibody-antigen interaction specificities were tested by inhibition ELISA. In inhibition ELISA, serum samples (1:100 dilutions) were co-incubated with varying concentrations of antigens (0–10 μg/mL) for 4 h at room temperature and overnight at 4°C. Then the reaction mixture was added to the antigen coated wells. The maximum percent of inhibition at 10 μg/mL for both antigens are presented in Figure 5 for all the samples. Inhibition against modified antigen yielded highest percent inhibition in breast cancer patients with depression (68.8% ± 7.8%) (Figure 5A), followed by prostate cancer patients with depression (62.9% ± 8.3%) (Figure 5B), breast cancer patients (48.9% ± 8.1%) (Figure 5C), and prostate cancer patients (43.4% ± 7.5%) (Figure 5D). Native antigen showed negligible inhibition in all the groups. Normal human subjects did not exhibit significant inhibition with any of the antigens (Figure 5E).

## 4 Discussion

Cancer disease has multifactorial etiology with concomitant involvement of several molecular components. Breast and prostate cancers are major cancer types in female and males, respectively. Cancer patients have faced several challenges during the unprecedented COVID-19 pandemic. Some of the major issues included non-availability of timely and specialized medical healthcare, limited availability of prescribed medications, and delays in follow-up procedures, etc. COVID-19 related depression further added to complications in such patients. Unnatural stress environment, which all the human beings were subject to during extended lockdown, social distancing, work from home policies extensively affected the mental health of individuals. These conditions induced anxiety and depression in large populations (Trabelsi et al., 2021; van der Werf et al., 2021). Depression was found to increase many-fold during the pandemic period, with associated negative impacts on cancer patients (Hussain et al., 2016; Bueno-Notivol et al., 2021).

Several factors may contribute to the development of cancers including lifestyle factors leading to hormone level imbalances, family history, exposure to carcinogens and pollutants, altered redox homeostasis, etc. (Anand et al., 2008). Cancer cells disrupt signaling pathways to alter oxidative metabolism (Schiliro and Firestein, 2021). Thus, there are increased levels of free radicals. Excessive free radicals have the potential to induce nucleic acid, protein and lipid degradation as well as post-translational modifications, leading to tumorigenesis (Wang et al., 2017). Serum samples of cancer patients in this study exhibited significantly high levels of oxidative stress markers such as MDA and carbonyl contents.

Cancer is a dangerous disease and patients are extremely fearful about its diagnosis and prognosis, causing high levels of distress (Smith, 2015). Long durations of anxiety and psychological distress in cancer patients lead to depression, with subsequent higher rates of mortality (Colleoni et al., 2000; Pinquart and Duberstein, 2010). One study showed various levels of depression can increase mortality rate by up to 39% (Satin et al., 2009). Considering these findings, we have designed this study, with two major types of cancer patients, i.e., breast and prostate alone or along with depression, for which clinical and immunological investigations were carried out to determine the impact and role of depression in such patients.

Cytokines play an important role in imbalance of oxidative levels and promote free radical production, which can alter DNA structure and lead to DNA mutations (Kartikasari et al., 2021). Furthermore, inflammatory cytokines are directly involved in epigenetic (Yasmin et al., 2015) and post-translational modifications (Louis and Bohjanen, 2017). In our results, cancer patients with depression exhibited higher levels of inflammatory cytokines (IFN-γ, TNF-α, and IL-6), as compared to the cancer patients without any depression. Other clinical factors (FBG, HbA1c, and BMI) were also analyzed, however, no remarkable changes were observed between patients and the healthy controls. An important inflammatory marker, CRP, was also found at significantly elevated levels in BC, PC, BCD, and PCD compared to NH. However, the levels of CRP were higher in breast and prostate cancer patients with depression as compared to BC and PC patients, respectively.

Plasmid DNA was modified using hydroxyl radical, and the structural alterations were observed using various biophysical characterizations (spectroscopy and fluorometry). Significant changes were found after the ^•^OH modification of pDNA, as compared to the native form.

Modified and native antigens were introduced into experimental animals to check their antigenicity. ^•^OH-pDNA exhibited a twofold higher antibody titre after eight doses of the antigen as compared to native pDNA. Antibody raised against ^•^OH-pDNA was found to be more specific to its antigen and gave 50% inhibition at lower concentrations as compared to its native form. These results showed ^•^OH-pDNA is more immunogenic and produced higher amounts of antibodies in animal model.

Previous studies showed the presence of autoantibodies against various antigens in cancer patients with depression (Khan et al., 2019b; Khan and Khan, 2020). In this study pDNA and ^•^OH-pDNA were used as antigens and cancer patients’ sera samples were screened for the presence of antibodies against these antigens. Direct binding ELISA showed, antigen ^•^OH-pDNA was recognized by higher levels of antibodies in cancer patients. Highest levels of anti-^•^OH-pDNA Abs were detected in breast cancer patients with depression followed by prostate cancer patients with depression. Comparatively, breast cancer and prostate cancer patients showed significantly lower levels of antibodies against the modified antigen. However, this level was still higher than the level of antibodies in normal human subjects. Serum anti-^•^OH-pDNA Abs were tested for their specificity using inhibition ELISA, which further ascertained direct binding results. Highest levels of percent inhibition were detected in BCD followed by PCD.

The findings from our study indicate that COVID-19 related depression in male (Prostate cancer) and female (breast cancer) cancer patients caused increased levels of oxidative stress, resulting in alterations in nucleic acid molecules. Additionally, higher levels of proinflammatory cytokines in depressed cancer patients further exaggerated destruction due to molecular alterations. Modified antigen showed high recognition of serum antibodies compared to the native antigen. Cancer patients with depression showed the higher levels of anti-^•^OH-pDNA Abs compared to the cancer patients without depression.

## 5 Conclusion

Cancer management has been extremely challenging during COVID-19 pandemic as there have been a lack of medical facilities and oncologists or physicians as well as limited availability of prescribed medications. Additionally, COVID-19 induced depression in a large population, including cancer patients. Increased serum levels of oxidative stress markers, inflammatory cytokines and an inflammatory marker were detected in BCD and PCD as compared to BC and PC patients. High levels of serum antibodies against ^•^OH-pDNA were detected in BCD and PCD as compared to BC and PC patients. Furthermore, inhibition ELISA also substantiated results of binding studies for the presence of antibodies against hydroxyl radical modified pDNA in BCD and PCD, as compared to BC and PC subjects. Thus, elevated levels of oxidative stress and inflammatory conditions, concomitant with depression in cancer patients cause increased generation of circulatory antibodies against autoantigens, which could lead to disease progression and development of additional complications. Thus, it is high time to address the impact of COVID-19 pandemic related depression on the mental health of cancer patients in an effort to provide focused medical care, treatment options and better overall disease management.

## Data Availability

The original contributions presented in the study are included in the article/supplementary material, further inquiries can be directed to the corresponding author.
